# Sexual Regional Dimorphism of Post-Adolescent and Middle Age Brain Maturation. A Multi-center 3T MRI Study

**DOI:** 10.3389/fnagi.2021.622054

**Published:** 2021-02-05

**Authors:** Giuseppe Delvecchio, Eleonora Maggioni, Alessandro Pigoni, B. Crespo-Facorro, Igor Nenadić, Francesco Benedetti, Christian Gaser, Heinrich Sauer, Roberto Roiz-Santiañez, Sara Poletti, Maria G. Rossetti, Marcella Bellani, Cinzia Perlini, Mirella Ruggeri, Vaibhav A. Diwadkar, Paolo Brambilla

**Affiliations:** ^1^Department of Pathophysiology and Transplantation, University of Milan, Milan, Italy; ^2^Department of Neurosciences and Mental Health, Fondazione IRCCS Ca' Granda, Ospedale Maggiore Policlinico, Milan, Italy; ^3^MoMiLab Research Unit, IMT School for Advanced Studies Lucca, Lucca, Italy; ^4^Department of Psychiatry, University Hospital Virgen del Rocío, IBiS, University of Sevilla, Sevilla, Spain; ^5^CIBERSAM, Centro Investigación Biomédica en Red Salud Mental, Santander, Spain; ^6^Department of Psychiatry and Psychotherapy, Philipps-University Marburg/Marburg University Hospital - UKGM, Marburg, Germany; ^7^Division of Neuroscience, Unit of Psychiatry and Clinical Psychobiology, IRCCS Ospedale San Raffaele, Milan, Italy; ^8^Department of Psychiatry, University Hospital Jena, Jena, Germany; ^9^Department of Psychiatry, School of Medicine, University Hospital Marqués de Valdecilla, University of Cantabria-IDIVAL, Santander, Spain; ^10^Department of Neurosciences, Biomedicine and Movement Sciences, Section of Psychiatry, University of Verona, Verona, Italy; ^11^Department of Neurosciences, Biomedicine and Movement Sciences, Section of Clinical Psychology, University of Verona, Verona, Italy; ^12^Department of Psychiatry and Behavioral Neurosciences, Wayne State University, Detroit, MI, United States

**Keywords:** sex differences, sexual dimorphism, aging, healthy individuals, magnetic resonance imaging

## Abstract

Sex-related differences are tied into neurodevelopmental and lifespan processes, beginning early in the perinatal and developmental phases and continue into adulthood. The present study was designed to investigate sexual dimorphism of changes in gray matter (GM) volume in post-adolescence, with a focus on early and middle-adulthood using a structural magnetic resonance imaging (MRI) dataset of healthy controls from the European Network on Psychosis, Affective disorders and Cognitive Trajectory (ENPACT). Three hundred and seventy three subjects underwent a 3.0 T MRI session across four European Centers. Age by sex effects on GM volumes were investigated using voxel-based morphometry (VBM) and the Automated Anatomical Labeling atlas regions (ROI). Females and males showed overlapping and non-overlapping patterns of GM volume changes during aging. Overlapping age-related changes emerged in bilateral frontal and temporal cortices, insula and thalamus. Both VBM and ROI analyses revealed non-overlapping changes in multiple regions, including cerebellum and vermis, bilateral mid frontal, mid occipital cortices, left inferior temporal and precentral gyri. These findings highlight the importance of accounting for sex differences in cross-sectional analyses, not only in the study of normative changes, but particularly in the context of psychiatric and neurologic disorders, wherein sex effects may be confounded with disease-related changes.

## Introduction

A vast literature (Ruigrok et al., 2014; Guebel and Torres, 2016; Marques et al., 2016; Gur and Gur, 2017), has come to recognize that the human brain, like many other biological organs, exhibits sexual dimorphism. An established finding is that men, on average, have a larger total brain volume compared to women, and the differences between sexes are reported in cortical and subcortical structures throughout the lifespan (Qiu et al., 2013; Ruigrok et al., 2014). The recent meta-analysis from Ruigrok et al. (2014) described several differences in brain volumes between males and females. Specifically, they observed that males showed higher gray matter (GM) volume in selective brain regions, including the amygdala, hippocampus, insular cortex, putamen, anterior parahippocampal gyri, posterior cingulate gyri, precuneus, temporal poles, and cerebellum, whereas females had higher GM volumes in the frontal pole, inferior and middle frontal gyri, pars triangularis, planum temporale/parietal operculum, anterior cingulate gyrus, insular cortex, and Heschl's gyrus (Ruigrok et al., 2014). These findings were partially confirmed in a more recent investigation that employed a single dataset with a very large sample of more than 5,000 healthy subjects (Ritchie et al., 2018). Indeed, the authors found that males generally showed larger regional volumes and cortical surface areas, while females appeared to be characterized by greater cortical thickness. After adjusting for global head measures, males showed greater volume in amygdala, putamen and in a set of cortical regions, with maximum difference in the left isthmus cingulate cortex, and an excess of surface area in more cortical regions. Conversely, females showed greater volume in cortical regions compared to males, with the greatest difference in favor of females in the right superior parietal cortex. Regarding the differences in white matter (WM), females showed lower fractional anisotropy and higher tract complexity compared to males. However, the complex relationship between sex and brain changes is yet to be fully understood, since maturational changes in the brain and many different factors, such as age and hormonal status, may interact with sex in the different stages of adult life.

These sex differences are hypothesized to begin in the perinatal and developmental phases of life, and indeed males typically show larger brains at birth, with increases in GM and WM volume, irrespective of head size (Gilmore et al., 2007). Moreover, sex-dependent trajectories determine differential timeframes of maturation between males and females. Most notably, during neurodevelopment, cerebral volume peaks earlier in females, but the rate of WM structural change is significantly greater in males (Lenroot et al., 2007; Tiemeier et al., 2010). Differences in the peak size of subcortical structures, such as the amygdala and the hippocampus, have also been observed in a sample ranging from 1 month to 25 years of age (Uematsu et al., 2012). These differential trajectories, which proceed throughout adolescence and adulthood following non-linear courses (Hedman et al., 2012), are suggested to be a product of an interaction between biology and the environment, with additional influences of sex chromosome genes, epigenetic mechanisms or sex hormones, acting via receptors throughout the developing and adult brain (McCarthy and Arnold, 2011; Lombardo et al., 2012; Koolschijn et al., 2014; Nguyen et al., 2017).

To date, however, most of the studies investigating sex effect on brain structures focused on samples in the early or late phases of their life (Gennatas et al., 2017; Ritchie et al., 2018). Indeed, only few investigations focused on the interaction between sex and age in the early and middle adulthood, usually thought as more stable periods (Guo et al., 2016). Understanding how sex differences impact on brain maturation is of crucial importance, as it may point toward the identification of sex-specific biological and environmental mechanisms that lead to the observed sex differences regarding both prevalence and features of mental diseases, such as psychiatric disorders (Venkatesh et al., 2008), and would also help understanding behavioral sex differences (Gur and Gur, 2017).

Thus far, although some studies assessed the interaction between sex and ages on brain structural changes during post-adolescence and middle adulthood, when disabling psychiatric disorders like schizophrenia begin to manifest (Immonen et al., 2017), the findings are still far to be conclusive. Indeed, given the differences between studies, results comparing such sex-age interactions during midlife (Guo et al., 2016) and between first and late adulthood (Jancke et al., 2015) are difficult to integrate. For instance, while the longitudinal study from Guo et al. (2016) reported differential regional brain changes between sexes (even after covarying for total brain volume changes), the cross-sectional study from Jancke et al. (2015) mostly attributed such differences to the influence of brain size. However, these inconsistencies might be due to the differences in the methodological approach employed by the individual studies. Indeed, Guo et al. (2016) employed a small but very well-characterized cohort while the study by Jancke et al. (2015) pooled together individuals from different cohorts, reaching a large but not homogenous sample. Moreover, neuroimaging studies (Taki et al., 2011; Ritchie et al., 2018) exploring the sex by age interaction effects on the brain reported significant differences in selective brain regions, especially in temporal, occipital (Taki et al., 2011), and frontal (Ritchie et al., 2018) lobes. However, also in this case, some heterogeneities have been observed between these studies. Indeed, the study by Ritchie et al. (2018) focused mostly on a sample of healthy subjects in their middle or late adulthood (mean age = 61 years old), phases where hormonal modifications, due to menopause (Zhang et al., 2016), might have acted as confounders, ultimately masking the real interaction between sex and age. In contrast, the study carried out by Taki et al. (2011) stratified their sample into decades, giving a broader picture spanning from early twenties to late sixties.

In view of the continuing uncertainty of the nature of sexual dimorphism on maturational changes in brain structure, our main aim was to investigate such dimorphism from the post-adolescence to middle adulthood phase, a period where the rate of neurobiological change is stable compared to other developmental phases, which are characterized by rapid changes in brain volumes (namely, adolescence and old age).

Using a structural magnetic resonance imaging (MRI) dataset of healthy subjects from the European Network on Psychosis, Affective disorders and Cognitive Trajectory (ENPACT) (Maggioni et al., 2017), we investigated sexual dimorphism in GM volume maturation trajectories by means of both voxel-based and region-based approaches, with the final goal of identifying (i) common and distinct patterns of brain structural changes throughout adulthood, (ii) differential areas of accelerated aging between sexes.

## Materials and Methods

### Participants

Structural MRI (sMRI) images from 385 left- and right-handed healthy individuals (191 females, 194 males, 30.38 ± 9.26 years, 18–62 years range) were used, being collected from four Clinical Research Centers adhering to the ENPACT network. Exclusion criteria included personal or family history of psychiatric illnesses, personal history of substance or alcohol abuse, intellectual disability or neurological disorders. All subjects provided a written informed consent to the protocol, which was drawn up in accordance with the Declaration of Helsinki and approved by the competent local Ethics committees. After visual inspection and quantitative quality check of the original and pre-processed images (described in section MRI Data Processing), 373 subjects were selected for the statistical analyses, including 183 females (30.82 ± 9.74 years) and 190 males (29.78 ± 8.52 years) without significant age differences (T = 1.09, *p* = 0.28). Based on the available information (364 subjects), education years were comparable between females (15.19 ± 2.91) and males (14.54 ± 3.55) (T = 1.9, *p* > 0.05). Details on sample demographics, MRI scanner type and acquisition parameters for each participating Center can be found in Table 1.

**Table 1:** ENPACT network demographics and image acquisition by Center.

**Site**	**Acronym**	**# females**	**# males**	**Age [years] females**	**Age [years] males**	**MRI scanner**	**MRI Sequence**	**Voxel Size (mm^**3**^)**	**Matrix Size**
Jena University Hospital, Jena, Germany	JHU	57	54	Mean: 30.37 SD: 8.94 Median: 27 Range: 20–55	Mean: 27.26 SD: 6.56 Median: 25 Range: 20–54	3D T1-weighted MPRAGE	3T Siemens Tim Trio scanner	1 × 1 × 1	256 × 256 × 192
University Hospital Marqués de Valdecilla, Santander, Spain	UHMV	43	62	Mean: 29.35 SD: 8.83 Median: 28 Range: 18–50	Mean: 28.76 SD: 7.05 Median: 27.5 Range: 19–49	3D T1-weighted FFE	3T Philips Intera scanner	0.94 × 0.94 × 1	256 × 256 × 160
University Vita-Salute San Raffaele, Milan, Italy	UVSSR	32	35	Mean: 35.31 SD: 12.63 Median: 29.5 Range: 22–60	Mean: 34.77 SD: 13.01 Median: 30 Range: 19–62	3D T1-weighted MPRAGE	3T Philips Gyroscan Intera scanner	0.9 × 0.9 × 0.8	256 × 256 × 220
University Hospital of Verona, Verona, Italy	VUH	51	39	Mean: 29.75 SD: 8.79 Median: 28 Range: 19–62	Mean: 30.44 SD: 6.11 Median: 29 Range: 21–46	3D T1-weighted MPRAGE	3T Magnetom Allegra Syngo Siemens scanner	1 × 1 × 1	256 × 256 × 160

*SD, standard deviation; MRI, magnetic resonance imaging; MPRAGE, Magnetization Prepared Rapid Acquisition Gradient Echo; FFE, Fast Field Echo*.

### MRI Data Processing

The entire dataset was processed at the University of Milan using the open-source Statistical Parametric Mapping software (version 12, https://www.fil.ion.ucl.ac.uk/spm) (Friston, 2003) running on Matlab R2018a (The Mathworks, Inc., Natick). Additional in-house Matlab scripts including functions from the Statistics and Machine Learning toolbox were developed to optimize the analyses. The Linux-based open-source Freesurfer image analysis suite (version 5.3.0, http://surfer.nmr.mgh.harvard.edu/) (Fischl, 2012) was also used for the preliminary intensity normalization step.

#### Pre-processing

The raw sMRI data from all subjects were visually inspected to identify images affected by gross movement or intensity inhomogeneity artifacts. All images were imported in Freesurfer and subjected to a Non-parametric Non-uniform intensity Normalization (N3) to correct for intensity inhomogeneity artifacts. As in Maggioni et al. (2017), this correction step was introduced to reduce artifactual intensity variations in the images (which can be acquisition-specific), thereby enhancing the accuracy and reliability of subsequent brain tissue segmentation for the multi-site dataset. The resulting images were then imported in SPM12 for voxel-based morphometry (VBM) analysis. The images were manually reoriented to align with the anterior-posterior commissure line on the horizontal axis. After a second bias field correction, the images were classified into GM and WM, cerebrospinal fluid (CSF), bone, soft tissue and air/background using SPM12 segmentation, which combines bias correction, tissue classification and registration to tissue probability maps in a single generative model. For each subject, the GM, WM, and CSF tissue maps in native space were converted into binary masks and integrated to estimate the subject's total intracranial volume (ICV). The subjects' GM and WM tissue class images were imported in the DARTEL (Diffeomorphic Anatomical Registration Through Exponentiated Lie algebra) toolbox. DARTEL estimates a non-linear deformation field for registering the GM and WM images of all participants. The non-linear registration procedure results in a group tissue map template and in warped versions of the tissue images aligned to the template. The registered GM images were normalized to the standard Montreal Neurological Institute (MNI) space via affine registration, spatially smoothed with a 3D 4 mm Full Width at Half Maximum (FWHM) Gaussian blurring kernel, and modulated using the Jacobian of the deformation field, which allows to preserve the original amount of signal from each voxel and to extract volume measures. A final quality control on the resulting GM images was performed using the VBM8 toolbox (Kurth et al., 2010) modules. After visual inspection of the registration across images, we assessed the image intensity homogeneity in terms of covariance and excluded the images with an overall covariance below two standard deviations. A group-specific optimal threshold GM mask was computed from the pre-processed images using the SPM masking toolbox (Ridgway et al., 2009).

#### Voxel-Based Design Specification and Statistical Inference

A voxel-wise GM volumetric comparison of age effects among sexes was performed using a mass-univariate General Linear Model (GLM) design in SPM12. Using a full factorial experimental design, the voxel-level GM volumes were modeled in terms of two main interacting factors: (a) Acquisition site (1. JUH, 2. UVSSR, 3. UHMV, 4. VUH) and (b) sex (1. females, 2. males). GM volume measures were assumed to be independent and with unequal variance between levels. Age was included as regressor of interest in the model and modeled as interacting with sex. The global nuisance effects of total tissue volumes were accounted for by dividing, subject by subject, the GM image intensities with the individual ICV value. Due to this proportional scaling, statistical inference was performed on the normalized GM volume measures. The group-specific optimal threshold GM mask was used as explicit mask in the analysis.

The GLM full factorial design specification was followed by the voxel-level estimation of the model coefficients via restricted maximum likelihood (REML) algorithm. The sex-specific effects of age on local GM volumes were assessed by making inference on the corresponding model coefficients with one-sided *t*-tests in both directions. In addition, a conjunction analysis was conducted to extract overlapping age-related changes in GM. In the former analyses, the significance threshold was set to *p* = 0.05 after peak-based Family Wise Error (FWE) multiple comparison correction, >200 voxels. Then, age by sex interactions were assessed through one-sided *t*-tests on the difference between the female- and male-specific age *beta* coefficients. Following an exploratory approach, the significance threshold was set to *p* = 0.001, >100 voxels. The choice of a less conservative cluster-based correction was motivated by the expected smaller size of the interaction effects compared to the main factor effects. The neuroanatomical location of the VBM clusters was defined using the Automated Anatomical Labeling (AAL) atlas (Tzourio-Mazoyer et al., 2002). Non-linear age by sex interaction effects on GM volumes were assessed in a secondary GLM analysis, which modeled age effects as quadratic (in interaction with sex).

#### Region-Based Volume Estimation, Design Specification, and Statistical Inference

Global and region of interest (ROI) analyses were performed to explore age by sex effects on global brain measures (total GM volume and ICV) and a priori defined anatomical regions (*N* = 116 AAL ROIs). Using Matlab in-house scripts, the GM volume in each AAL ROI was estimated by a summation of the GM density values of the voxels within the ROI. Total GM volume was calculated as the sum of AAL ROI volumes. The AAL ROI and total GM volumes were used as response variables in GLM designs that included sex, center, age and ICV as predictors, with age and sex interacting. In ICV analysis, ICV was modeled in terms of sex, center, and age (interacting with sex). As in the VBM analysis, we ran a secondary ROI GLM analysis that considered quadratic age effects (in interaction with sex). For each ROI, the model coefficients were estimated through least-squares fitting of the link between the response variable (ROI volume) and the model predictors. Inference on the interaction between age and sex was performed through two-sided *t*-tests on the GLM coefficient relative to the product of age and sex predictors. Significance was set to *p* = 0.05, with and without multiple comparison correction based on the Bonferroni's method with N equal to the number of AAL ROIs.

## Results

### Voxel-Based Morphometry

**Figure 1:**
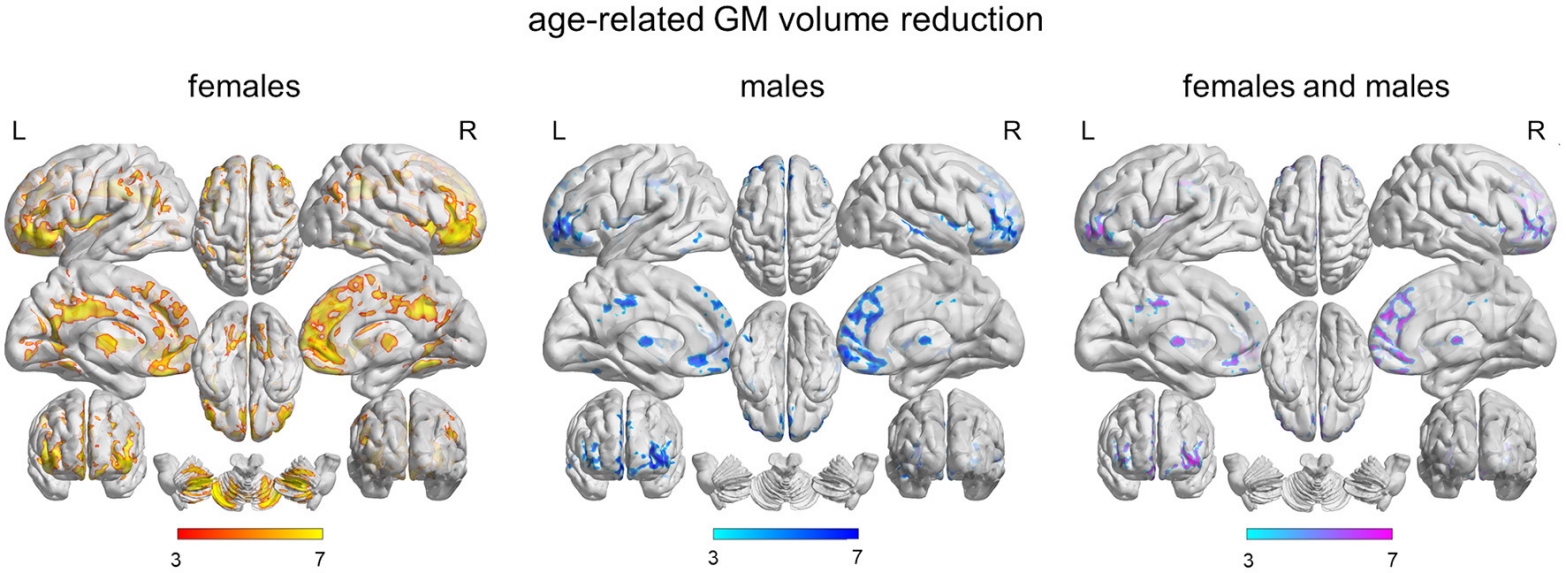
VBM results, age-related GM volume changes. Brain clusters showing significant GM volume reduction during adulthood in females (left panel), males (central panel), and in both genders (right panel) (*p* < 0.05, pFWE, >200 voxels). VBM, voxel-based morphometry; GM, gray matter; pFWE, peak-based family wise error.

The brain clusters showing significant GM volume changes with age, in females and males separately, are illustrated in Figure 1, left and central panels. The neuroanatomical labeling, extension, and statistical details of the VBM cluster peaks are reported in Tables 2A,B. No age-related GM volume increases emerged in either sexes (*p* < 0.05, pFWE, >200 voxels). In females, aging was associated with spread bilateral GM volume reductions that involved the frontal cortex, especially the superior medial portion, the cingulate and insular cortices, the precentral and postcentral gyri and the temporo-parieto-occipital region, especially the angular gyri (*p* < 0.05, pFWE, >200 voxels). Bilateral age-related GM volume reductions emerged also at the subcortical level, in the putamen and caudate and in a wide portion of the cerebellum. In males, aging was associated with bilateral GM volume decreases in the frontal cortex, especially in mid orbitofrontal gyrus, left inferior and superior temporal gyri, right middle temporal gyrus, insular and mid cingulate cortices and thalami (*p* < 0.05, pFWE, >200 voxels). The conjunction analysis showed that females and males experienced common age-related GM volume reductions in most of the frontal clusters emerged from the separate analyses of females and males, and in the bilateral thalami, insular, and mid cingulate cortices (*p* < 0.05, pFWE, >200 voxels) (Table 2C and Figure 1, right panel).

**Table 2:** Sex-specific and conjunction VBM results.

	**Contrast**	**# Voxels**	**P (pFWE)**	**Peak T**	**X, Y, Z (mm)**	**AAL region**
Sex-specific analysis	A. Age negative, females	53,400	<0.001	9.68	51, 32, −9	Inferior orbitofrontal, R
		225	<0.001	7.76	−45, −44, 53	Inferior parietal, L
		932	<0.001	7.70	−53, −63, 26	Angular, L
		346	<0.001	7.67	36, −2, 57	Mid frontal, R
		273	<0.001	7.49	−56, −9, 38	Postcentral, L
		503	<0.001	7.44	45, −39, 48	Inferior parietal, R
		625	<0.001	7.26	−2, −6, 5	Thalamus, L
		533	<0.001	7.22	51, −66, 29	Mid occipital, R
		413	<0.001	7.19	48, −14, 42	Precentral, R
		259	<0.001	7.17	−6, 18, 6	Caudate, L
		403	<0.001	7.05	11, 3, 20	Caudate, R
		233	<0.001	6.87	47, 12, 29	Inferior frontal, opercular, R
		423	<0.001	6.84	−2, −81, −5	Calcarine, L
		1,080	<0.001	6.59	−15, −56, −54	Cerebelum 8, L
	B. Age negative, males	9,555	<0.001	8.33	−39, 56, 0	Mid orbitofrontal, L
		437	<0.001	7.28	63, −29, −3	Mid temporal, R
		734	<0.001	6.98	44, 6, 3	Insula, R
		940	<0.001	6.90	−39, −5, 11	Insula, L
		846	<0.001	6.79	−6, −24, 45	Mid cingulum, L
		351	<0.001	6.69	−50, −60, −17	Inf temporal, L
		216	<0.001	6.36	0,−9, 8	Thalami
		252	<0.001	6.33	−42, −8, −9	Sup temporal, L
Conjunction analysis	C. Age negative, males and females	5,894	<0.001	7.95	−38, 56, 0	Mid orbitofrontal, L
		821	<0.001	7.20	3, 39, −11	Mid orbitofrontal, R
		629	<0.001	6.89	−39, −5, 11	Insula, L
		503	<0.001	6.71	8, 32, −21	Rectus, R
		651	<0.001	6.67	0, −27, 41	Mid cingulum
		584	<0.001	6.67	41, 8, 2	Insula, R
		210	<0.001	6.36	0, −9, 8	Thalami

*VBM, voxel based morphometry; x, y, z (mm), coordinates in MNI space; T, T-statistics; P(pFWE), p-value, peak-based family wise error corrected; AAL, Automated Anatomical Labeling*.

**Figure 2:**
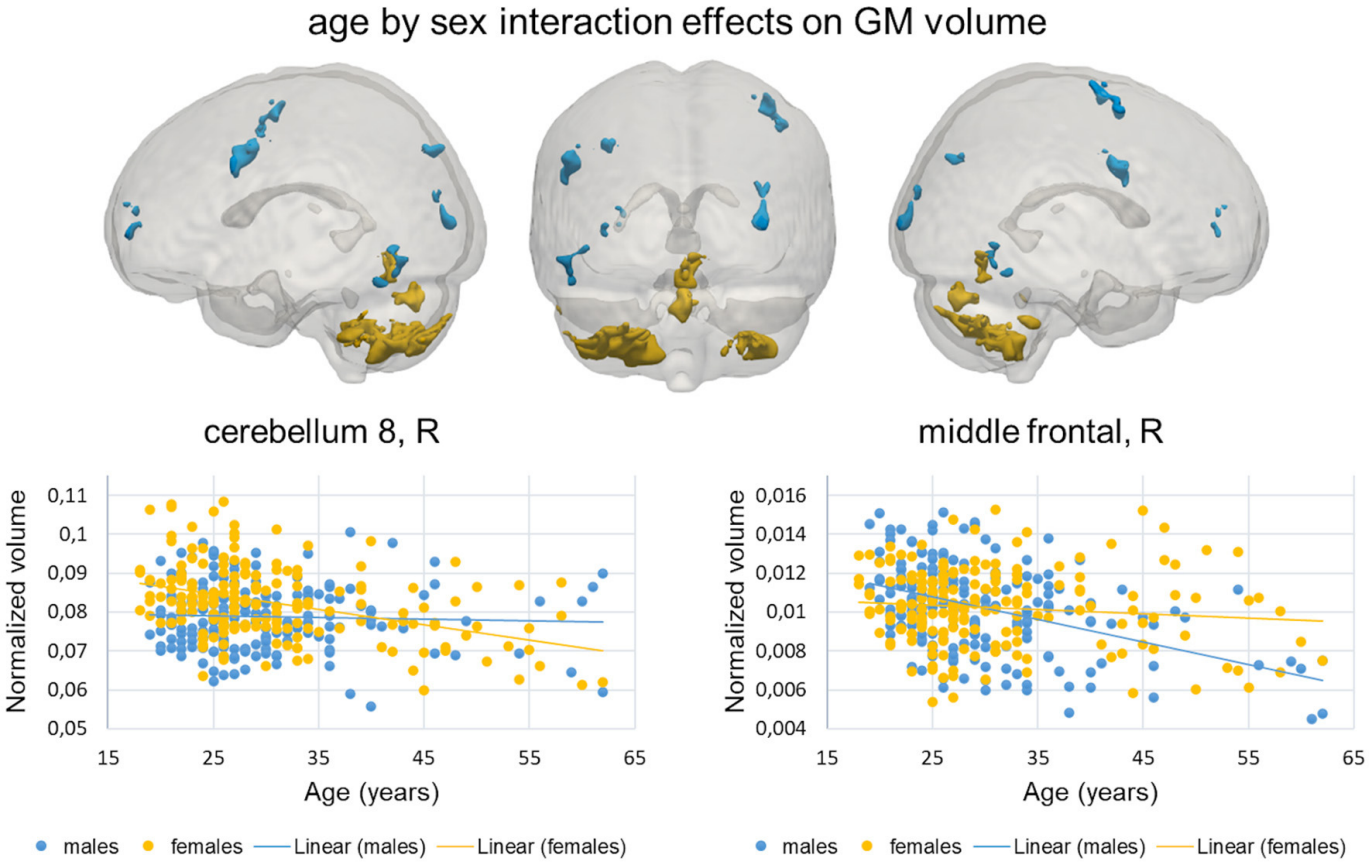
VBM results, age by sex interactions. Top: Brain clusters showing significant age by sex interaction effects (*p* ≤ 0.001, >100 voxels). The clusters with enhanced age-related changes in females and males are represented in orange and cyan colors, respectively. Bottom: scatterplots showing normalized GM volume changes with aging in females (orange) and males (cyan) in two exemplar clusters.

The age by sex interaction analysis identified a set of brain clusters with different age-related GM volume changes between the two sexes (*p* ≤ 0.001, >100 voxels), which are shown in Figure 2. Females showed enhanced age-related GM volume reductions in bilateral portions of the cerebellum and vermis compared to males (Table 3A, orange clusters in Figure 2). Conversely, males exhibited higher age-related GM volume reductions in bilateral mid frontal and occipital cortices and in left precentral and inferior temporal gyri (Table 3B, cyan clusters in Figure 2). The scatterplots in Figure 2 illustrate the relation between age and GM volume in the clusters within the right cerebellum and right mid frontal cortex, showing the most salient age-related changes in females (*vs*. males) and males (*vs*. females), respectively. The secondary non-linear GLM analysis, which modeled age effects as quadratic instead of linear, showed interaction effects in the same brain regions (results not shown).

**Table 3:** Age by sex interactions from VBM analysis and ROI analysis on VBM clusters.

**VBM contrast**	**# Voxels**	**VBM peak**			
		**x, y, z (mm)**	**AAL region**	**T**	**P (unc)**
A. Age negative, females > males	755	24, −60, −44	Cerebelum 8, R	5.51	<0.001
	1,766	−27, −65, −44	Cerebelum 8, L	5.09	<0.001
	121	−57, −45, −38	Cerebelum crus 1, L	4.59	<0.001
	275	6, −63, −9	Vermis 4	3.93	<0.001
	292	5, −78, −24	Vermis 7 and 8	3.32	<0.001
B. Age negative, males > females	237	48, 2, 56	Mid frontal cortex, R	3.65	<0.001
	114	−29, −75, 41	Mid occipital cortex, L	3.57	<0.001
	298	−53, 5, 36	Precentral gyrus, L	3.50	<0.001
	121	−35, 57, 14	Mid frontal cortex, L	3.34	<0.001
	165	33, −92, 24	Mid occipital cortex, R	3.19	=0.001
	145	−50, −56, −21	Inferior temporal gyrus, L	3.14	=0.001

*VBM, voxel based morphometry; ROI, region of interest; x, y, z (mm), coordinates in MNI space; T, T-statistics on age by sex interaction contrast; P (unc), p-value, uncorrected; P (Bonf), p-value after Bonferroni's correction (N = 11); AAL, Automated Anatomical Labeling; n.s., not significant*.

### Region of Interest Analysis

**Figure 3:**
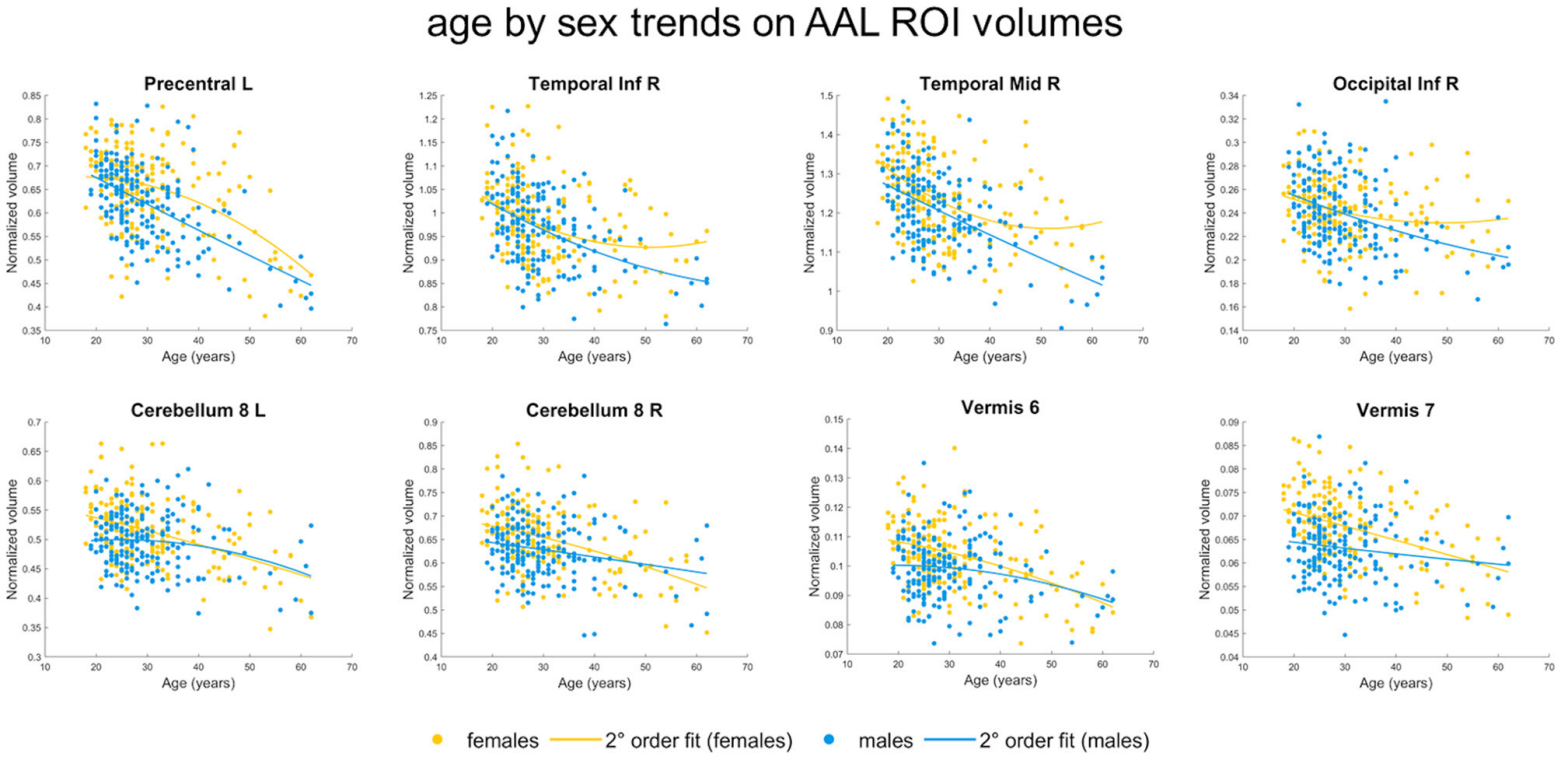
ROI results, age by sex interactions. Scatterplots (and 2nd order fits) showing normalized GM volume changes with aging in females (orange) and males (cyan) in the AAL ROIs with age by sex trend effects (*p* < 0.05). ROI, region of interest; AAL, Automated Anatomical Labeling.

Total GM volume and ICV did not exhibit age by sex effects (*p* < 0.05). The assessment of age by sex interactions on the AAL ROI volumes showed no results after Bonferroni's correction (*N* = 116), but some trends emerged (Table 4, section “Linear age model”). In females, aging was associated with more pronounced GM volume reductions than in males in the bilateral VIII lobule of the cerebellar hemisphere and in lobules VI and VII of the vermis. Conversely, in males, aging induced enhanced GM volume reductions compared to females in left precentral gyrus, inferior and middle portions of right temporal cortex and right inferior occipital cortex. All these ROIs except from the left precentral gyrus exhibited age by sex trend effects also when quadratic age effects were modeled instead of linear ones (Table 4, section “Quadratic age model”). The emerged tendencies can be appreciated by looking at Figure 3 scatterplots, which show age-related GM volume changes in these AAL ROIs, for females and males separately.

**Table 4:** Age by sex interactions from ROI analysis on AAL atlas regions.

**AAL region**	**Linear age model**			**Quadratic age model**			**Age by sex interaction side**
	**T**	**P (unc)**	**P (Bonf)**	**T**	**P (unc)**	**P (Bonf)**	
Cerebelum 8, L	2.22	0.027	n.s.	2.08	0.038	n.s.	Enhanced age effects in females
Cerebelum 8, R	2.05	0.041	n.s.	2.12	0.034	n.s.	
Vermis 6	2.19	0.029	n.s.	2.17	0.031	n.s.	
Vermis 7	2.50	0.013	n.s.	2.49	0.013	n.s.	
Precentral, L	2.10	0.037	n.s.	1.66	n.s.	n.s.	Enhanced age effects in males
Inf occipital, R	2.49	0.013	n.s.	2.44	0.015	n.s.	
Mid temporal, R	2.34	0.020	n.s.	2.5	0.013	n.s.	
Inf temporal, R	2.21	0.028	n.s.	2.2	0.027	n.s.	

*T, T-statistics on age by sex interaction contrast; P (unc), p-value, uncorrected; P (Bonf), p-value after Bonferroni's correction (N = 116); ROI, region of interest; AAL, Automated Anatomical Labeling; n.s., not significant*.

## Discussion

Life is change and change is an immutable characteristic of the lifespan of an individual, at both neurobiological and behavioral levels. Interestingly, neuroanatomical changes seem to occur also in phases of life considered stable, as suggested by our study, which identified age-related GM volume changes in a multi-site sample of healthy individuals in their early-to-middle adulthood.

In the present study, voxel-based and region-based approaches were used in concert to provide a detailed assessment of the patterns of sexual dimorphism. Both sets of analyses suggest that patterns of sexually dimorphic maturational changes endure even in a relatively stable phase of the life span, such as young and middle adulthood (Guo et al., 2016). These findings are not surprising, especially because sex differences in brain structure have been consistently observed at different lifetime phases, including infancy (Knickmeyer et al., 2014), adolescence (Gennatas et al., 2017) and late adulthood (Ritchie et al., 2018). Thus, our results further highlight that the brain volume is continuously changing, with sexual dimorphism being more evident in some regions than others.

### Age-Related GM Patterns in Females

In females, aging was associated with bilateral GM volume reductions in cortical, including frontal, cingulate, insular cortices and the temporo-parieto-occipital junction, and subcortical, especially putamen and caudate, as well as cerebellar, areas. Overall, these results aligned with the evidence reported by previous literature showing selective GM volume deficits in female subjects in healthy populations (Ritchie et al., 2018).

Notably, in our results, the more diffused age-related reductions in females were located in areas considered key regions for emotion recognition and processing, such as the temporo-parietal junction (Lettieri et al., 2019), which are part of the fronto-limbic pathway where sex-dependent differences were often observed (Kong et al., 2014; Lungu et al., 2015). A recent study (Abbruzzese et al., 2019) suggested that emotion recognition might be influenced by both age and gender and linked such differences to differential cognitive abilities and exploration strategies among sexes. These findings could be attributed to differences between men and women in sex steroid hormones, as many studies reported a link between emotional processing and testosterone, estrogen, and progesterone (Bos et al., 2013; Toffoletto et al., 2014).

Importantly, our findings might be of interest also for clinical psychiatric populations, given that the post-adolescence period is critical for such diseases' onset (Kelly et al., 2016). Also, strong sex-dependent differences are well described in major depression in terms of prevalence, symptoms (Salk et al., 2017), and neurobiological alterations (Yang et al., 2017; Jenkins et al., 2018), with females resulting more affected than males. Therefore, increasing our understanding of how brain structures evolve through post-adolescence and middle adulthood in healthy subjects might help in further describe the onset and progression of psychiatric disorders.

Similarly, sex differences have been often reported in the incidence of late-life neurological disorders, including dementia, where being a woman is considered one of the major risk factors for late-onset Alzheimer disease (Farrer et al., 1997; Ferretti et al., 2020). Moreover, these sex-specific effects were more pronounced in women in peri- and, even more, in post-menopause, suggesting a role of sex hormones and aging. However, such differences might arise earlier in the life span, and years before menopausal hormonal changes. In conclusion, although these results need to be confirmed by longitudinal studies, they might represent a first step in a more detailed understanding of how our brain ages through the years, thus opening new possibilities for the understanding of neurodevelopmental disorders and prevention of neurodegenerative diseases.

### Age-Related GM Patterns in Males

In males, aging was associated with bilateral GM volume reductions in the frontal cortex, in the three major temporal gyri, insular, and mid cingulate cortices. Also in this case, these results are in line with recent evidence reporting a steeper volume decline in males in temporal regions (Ritchie et al., 2018). One potential explanation of such specificity is the effect, in specific areas, of sex-biased gene expression (Kang et al., 2011) or other protective genetic or hormonal factors that might “buffer” females from potential brain volume alterations, especially against neurodevelopmental disorders (Jacquemont et al., 2014). Among those, one candidate is the hypothesized “female-protective” mechanism that involves a major genetic stability against mutations in females, conferred by the second copy of the X chromosome (Reinhold and Engqvist, 2013).

Notably, frontotemporal areas showed also great sexual dimorphism in fMRI studies conducted on emotional tasks (Hofer et al., 2007; Repple et al., 2018). Specifically, prefrontal regions are involved in the top-down emotional control and a recent study on healthy subjects pointed out the differential role of these areas in men and women as a possible neurobiological mechanism explaining the different response between sexes to anger and aggressive impulses, usually considered more “manly” behaviors (Repple et al., 2018). Moreover, both prefrontal and temporal areas present sex-driven functional and structural asymmetry (Hofer et al., 2007; Guadalupe et al., 2015) and temporal gyri have been also described among the most dimorphic areas of the brain, showing one of the strongest sex-linked asymmetries, especially in males.

Finally, the possibility that the healthy brain might also help us in shedding a light on brain disorders is also valid for the results observed in our group of male subjects. Indeed, GM abnormalities in the temporal gyri have been often associated with psychotic syndromes, especially with schizophrenia (Ohi et al., 2016; Delvecchio et al., 2017; Squarcina et al., 2017), which is a disorder where sexual dimorphism is well-described, with male individuals showing earlier onset, more severe course of the illness, and poorer long-term prognosis compared to matched females (Kelly et al., 2016).

Therefore, overall these findings point toward the hypothesis that temporal regions might represent regions of specific fragility in males.

### Differences in Age-Related GM Changes Between Males and Females

Voxel-based and region-based analyses were combined to identify age by sex interaction effects on GM volume. The results showed that females had enhanced age-related GM volume loss in bilateral portions of the cerebellum and vermis, while males exhibited higher age-related GM volume loss in portions of frontal, occipital, and temporal gyri. Our findings are in line with previous literature showing sex differences in cerebellum and frontotemporal areas (Ruigrok et al., 2014; Ritchie et al., 2018), with males usually showing greater volumes in cerebellum, while females having greater volumes in frontal regions.

The finding regarding cerebellum is especially interesting. Far from being just a regulator of movement and balance, this structure is known to have roles in cognition and emotions in healthy individuals (Hofer et al., 2007). Moreover, recent literature suggested that the cerebellum may play a key role in modulating cognitive dysfunctions in Alzheimer (Jacobs et al., 2018), a typically female disease (Ferretti et al., 2020). The widespread cognitive deficits observable in Alzheimer patients seem to be under cerebellar modulation and dependent from cerebro-cerebellar circuits linked with the cerebral associative and paralimbic regions (Jacobs et al., 2018). Therefore, the enhanced reduction of cerebellar GM volume in relation to age observed in our female subjects might suggest a specific vulnerability for neurodegenerative diseases, rooted in sex-dependent differences in brain maturation.

Finally, it is important to point out that sex differences in brain structures are a product of the interplay of biological and environmental influences on brain development (McCarthy and Arnold, 2011). Animal and human studies have shown the influence of steroid hormones, sex chromosomes (Arnold and Chen, 2009), and the immune system (Lenz et al., 2013) on the development of neural sexual differentiation, starting from the prenatal period. In addition, environmental factors, such as stress and maternal infections (Bale et al., 2010), and postnatal factors, such as early childcare, have been also found to influence brain development (Giedd et al., 2012). As it was hypothesized (Pletzer, 2019), testosterone and estradiol/progesterone might differentially affect GM volumes in areas that are usually larger in males, such as hippocampus or cerebellum, or in females, such as the frontal lobe (Ruigrok et al., 2014; Ritchie et al., 2018). In this regard, our results seem to confirm such hypothesis, with females showing a greater effect of age on cerebellum and males on frontotemporal areas. Moreover, our results highlight how the brain is in constant remodeling throughout adulthood, even in a period of relative stability (Guo et al., 2016). This notion is in line with the literature on developmental sex differentiation of the brain that suggested the impact of genes on sex hormones both during the development and in the adult's brain (McEwen and Milner, 2017).

### Limitations and Future Perspectives

While evaluating the presented results, some limitations should be considered. The major limitation to our study is its cross-sectional design. Longitudinal studies—able to assess the reduction in GM volumes over time and the association of brain maturation trajectories with the onset of psychiatric and neurodegenerative disorders—are needed to understand the deep and intertwined effects of sex and age. The unequal distribution of subjects within the considered age range, with the majority of subjects in the early adulthood phase, might have limited the power of identifying age differences within and between sexes, undermining the age by sex interaction effects in some brain regions. On the other hand, this unbalance might have reduced the confounding effects of menopause or associated hormone replacement therapy on the brain (Zhang et al., 2016). Age by sex influences on local GM volume mainly emerged at the trend level, thus need to be reproduced on independent datasets. The use of a linear model design might have undermined the identification of more complex relations between the predictor variables, including age and sex, and local GM volumes. At the same time, this essential design facilitated the extraction and interpretation of the complex interaction effects. Future analyses using more flexible approaches, including smooth non-parametric regression models, are planned to confirm and extend the obtained results. A further extension is represented by surface-based analyses, both vertex- and region-wise, which provide a complementary perspective of age by sex effects on GM morphology. Handedness was not considered as an inclusion/exclusion criterion. Therefore, although the effect of handedness on brain morphology is controversial, we cannot rule out its impact on structural brain changes observed in our group of healthy individuals. Finally, no behavioral measures were available for our sample and therefore we could not correlate brain alterations with behavioral data to have a clearer picture of how age and sex shape brain structure and function and in turn behavior.

## Conclusions

As neurological and psychiatric research moves toward the ideals of precision medicine (Pigoni et al., 2019), it is getting everyday more necessary to have a clear, nuanced understanding of similarities and differences in brain structures and functions across the sexes. The potential effects of genes and hormones on brain sex differences are likely active at multiple points across the lifespan, representing a constant set of influences that interact with the environment in a complex and intertwined manner. For these reasons, it is of great importance to study sex differences in human brain at different developmental phases and to explore how they interact with normal and pathological aging. Indeed, a better comprehension of how the brain ages in post-adolescence and middle-adulthood and the different trajectories between males and females might help us in disentangling the different expression of psychiatric and neurologic disorders that interact to a higher extent with sexual dimorphic aspects in specific phases of life. Specifically, our results corroborate the notion that the interaction between age and sex occur even a relatively stable period of life, from early to middle adulthood. Finally, despite the presence of heterogeneities, especially in terms of enrollment and methods employed (e.g., strength and type of MRI scanner, voxel-based vs. ROI-based approaches), these findings might prove important while studying the neural basis of the known differences between males and females in the risk of developing psychiatric and neurologic disorders.

## Data Availability Statement

The raw data supporting the conclusions of this article will be made available by the authors, without undue reservation.

## Ethics Statement

The studies involving human participants were reviewed and approved by local research ethics committees. The patients/participants provided their written informed consent to participate in this study.

## Author Contributions

PB: conceptualization, funding acquisition, supervision, and writing—review and editing. GD: writing-original draft and supervision. EM: writing-original draft, data curation, methodology, and formal analysis. AP: writing-original draft. BC-F, IN, FB, CG, HS, SP, MR, MB, CP, and MGR: data curation and writing—review and editing. VAD: conceptualization, supervision, and writing—review and editing. All authors contributed to the revision process of the manuscript and approved the final version of it.

## Conflict of Interest

The authors declare that the research was conducted in the absence of any commercial or financial relationships that could be construed as a potential conflict of interest.
